# Multi-Regional Adaptation in Human Auditory Association Cortex

**DOI:** 10.3389/fnhum.2017.00247

**Published:** 2017-05-09

**Authors:** Urszula Malinowska, Nathan E. Crone, Frederick A. Lenz, Mackenzie Cervenka, Dana Boatman-Reich

**Affiliations:** ^1^Departments of Neurology, Johns Hopkins School of Medicine, BaltimoreMD, USA; ^2^Department of Neurosurgery, Johns Hopkins School of Medicine, BaltimoreMD, USA; ^3^Department of Otolaryngology, Johns Hopkins School of Medicine, BaltimoreMD, USA

**Keywords:** auditory cortex, adaptation, high-gamma, repetition suppression

## Abstract

In auditory cortex, neural responses decrease with stimulus repetition, known as adaptation. Adaptation is thought to facilitate detection of novel sounds and improve perception in noisy environments. Although it is well established that adaptation occurs in primary auditory cortex, it is not known whether adaptation also occurs in higher auditory areas involved in processing complex sounds, such as speech. Resolving this issue is important for understanding the neural bases of adaptation and to avoid potential post-operative deficits after temporal lobe surgery for treatment of focal epilepsy. Intracranial electrocorticographic recordings were acquired simultaneously from electrodes implanted in primary and association auditory areas of the right (non-dominant) temporal lobe in a patient with complex partial seizures originating from the inferior parietal lobe. Simple and complex sounds were presented in a passive oddball paradigm. We measured changes in single-trial high-gamma power (70–150 Hz) and in regional and inter-regional network-level activity indexed by cross-frequency coupling. Repetitive tones elicited the greatest adaptation and corresponding increases in cross-frequency coupling in primary auditory cortex. Conversely, auditory association cortex showed stronger adaptation for complex sounds, including speech. This first report of multi-regional adaptation in human auditory cortex highlights the role of the non-dominant temporal lobe in suppressing neural responses to repetitive background sounds (noise). These results underscore the clinical utility of functional mapping to avoid potential post-operative deficits including increased listening difficulties in noisy, real-world environments.

## Introduction and Background

Neural responses in sensory cortex decrease with stimulus repetition, known as adaptation or repetition suppression (Li et al., 1993; Grill-Spector et al., 2006). In auditory cortex, adaptation is thought to enhance detection of novel sounds and facilitate listening in noisy environments (Ulanovsky et al., 2003; Taaseh et al., 2011). Adaptation has been studied in animal primary auditory cortex using pure tones (Condon and Weinberger, 1991; Ulanovsky et al., 2003, 2004; Wehr and Zador, 2005; Taaseh et al., 2011). Primary auditory cortex is tonotopic and corresponds to Heschl’s gyrus, the first cortical region to receive auditory input. Scalp EEG studies have confirmed rapid adaptation to tones in human cortical auditory areas (Costa-Faidella et al., 2011; Herrmann et al., 2013; Lanting et al., 2013). Although the high temporal resolution of scalp recordings is well suited for capturing the rapid changes in neural activity (ms), poor spatial resolution has precluded precise localization within auditory cortex.

Intracranial electrocorticography (ECoG) is performed in epilepsy patients who are surgical candidates. ECoG signals are recorded directly from cortex, providing high spatial-temporal resolution and robust sampling of high-gamma (HG) frequencies (>60 Hz) implicated in sensory perception (Bertrand and Tallon-Baudry, 2000). Event-related increases in HG power are largely non-phase locked and thought to reflect local neuronal population activity (Mukamel et al., 2005; Ray et al., 2008; Ray and Maunsell, 2011). ECoG electrodes are often implanted over perisylvian cortex, including auditory association areas on the lateral superior temporal gyrus. Auditory association cortex receives input from primary auditory cortex and is implicated in processing complex sounds as an intermediate stage along the hierarchical auditory processing pathway (Blumstein et al., 1977; Boatman et al., 1995; Rauschecker et al., 1995; Howard et al., 2000; Kaas and Hackett, 2000; Miglioretti and Boatman, 2005). Previous ECoG studies have shown robust HG responses to speech in auditory association cortex (Crone et al., 2001; Sinai et al., 2009; Chang et al., 2010; Steinschneider et al., 2011; Mesgarani et al., 2014). HG responses in auditory association cortex also show repetition-related adaptation (Eliades et al., 2014) and increased local network activity as measured by low-frequency (4–7 Hz; theta) coupling (Malinowska and Boatman-Reich, 2016).

It is not known, however, whether adaptation occurs locally in auditory association cortex or originates from primary auditory cortex. Resolving this issue is important for advancing our understanding of cortical adaptation and to identify potential post-operative deficits that could affect the every-day listening abilities of patients undergoing temporal lobe resections (Drew et al., 2003; Nagle et al., 2013). Determining the neural bases of adaptation also has implications for functional neuroimaging (fMRI) studies that use adaptation paradigms to investigate sub-regional functional selectively (Kar and Krekelberg, 2016).

It has been difficult to resolve this issue because ECoG electrodes are rarely implanted directly in primary auditory cortex (Howard et al., 1996, 2000). We had a rare opportunity to study a patient with electrodes implanted in primary and association auditory cortex for clinical purposes. By recording simultaneously from both regions it was possible to directly compare temporal-spatial patterns of adaptation. We hypothesized that adaptation to simple tones occurs in primary auditory cortex, whereas adaptation to complex speech and non-speech sounds originates in auditory association cortex.

## Materials and Methods

### Subject

A 47-year-old right-handed man, a self-employed college graduate, was referred for surgical evaluation for medically intractable, daily complex partial seizures that began at age 16. His history was negative for febrile seizures, head trauma or familial seizures. Scalp EEG showed focal slowing over right parietal cortex; MRI revealed small focal dysgenesis (∼1 cm) in the right inferior parietal lobe, posterior to the post-central sulcus. His hearing thresholds (≤25 dB HL) and cognitive function (FSIQ 109) were normal. Speech recognition was normal in quiet and noise (SCAN-A Test, Pearson Corp; BKB SIN test, Etymotic Research). Prior fMRI studies confirmed left hemisphere language dominance. The patient provided informed written consent in accordance with Johns Hopkins Institutional Review Board requirements.

### Electrode Implantation

Subdural electrodes were implanted based on localization of the right parietal dysgenesis. A grid of 8 × 8 macro-electrodes (2.3 mm diameter, 1-cm spacing) was implanted over lateral, right temporal and parietal cortex (Ad-Tech, Racine, WI, USA). Two depth electrodes, with five evenly spaced cylindrical contacts at the distal ends (0.8 mm × 2.5 mm; 2 mm spacing), were inserted through small incisions in the grid: one posterior and one anterior to the region of dysgenesis (Supplementary Figure 1). The posterior depth (DP) was inserted at the level of the Sylvian fissure, orthogonal to the convexity of the superior temporal gyrus and inferior and posterior to the dysgenesis. The second depth (DA) was implanted in inferior parietal cortex, anterior to the dysgenesis. Electrode locations were determined by co-registration of post-surgical CT scans with pre-surgical 3D MRI and intraoperative photographs using BioImage Suite (Duncan et al., 2004). Location of co-registered depth and grid electrodes was normalized to a standardized brain map, the Montreal Neurological Institute (MNI) brain atlas, and assigned 3D coordinates (Papademetris et al., 2006). MNI coordinates were converted to Talairach coordinates and assigned a corresponding Brodmann area (Lacadie et al., 2008).

### Stimuli and Task

#### Stimuli

Six auditory stimuli of 200 ms duration (5-ms rise/fall) were used to generate three pairs of: (1) steady-state tones (1000 Hz, 1200 Hz; NCH Tone/Waveform Generator; NCH Software); (2) frequency-modulated (FM) tones with linear sweeps (upward, downward) from 800 Hz or 1600 Hz to a 1200 Hz target; and (3) digitized speech syllables (/ba/, /da/) (44.1 kHz, 16 bit sampling; Sound Forge, Sony). The two tone frequencies have been used in prior auditory oddball studies (Todorovic et al., 2011; Cervenka et al., 2013). For consistency, FM tone and speech pairs were differentiated along a single acoustic-phonetic parameter (sweep direction, consonant place-of-articulation). All stimulus pairs are readily discriminated by normal listeners and were presented binaurally at comfortable listening levels through insert earphones (ER2, Etymotic Research, Elk Grove Village, IL, USA).

#### Task

Auditory stimuli were presented in a 300-trial passive oddball paradigm. Oddball paradigms are useful for studying adaptation because one stimulus is repeated consecutively and frequently (adapting), interspersed by a second, infrequent stimulus (Näätänen et al., 1982; Escera et al., 1998; Ulanovsky et al., 2004). We chose passive listening to avoid attentional effects and for consistency with prior adaptation studies. One stimulus from each pair was repeated in trains of 2-12 consecutive trials (1000 Hz, upward FM tone, /ba/); the other was presented infrequently and non-consecutively using a Neuroscan STIM 2 system (Compumedics Inc., El Paso, TX, USA). The inter-stimulus interval was 1200 ms, within the 1000–1800 ms range considered sufficiently long to avoid overlap between the neural response and the next stimulus presentation, while sufficiently short to elicit adaptation (Edwards et al., 2005; Taaseh et al., 2011). The patient was instructed to ignore the auditory stimuli and attend to a silent animated movie. Recordings were completed over two sessions in 1 day.

### ECoG Recordings

Recordings were performed at the bedside 4 days after electrode implantation while the patient was awake. Antiepileptic medications had been discontinued for seizure localization. Continuous ECoG was acquired simultaneously from all electrodes using a 128-channel Stellate System (Montreal, QC, Canada). ECoG signals were amplified (5 × 1000) and recorded digitally using a referential montage, 1000 Hz A/D sampling, and a bandpass filter of 0.03–250 Hz (6 dB/octave). The reference electrode was at the top right corner of the grid. Stimulus onset markers were recorded simultaneously to separate EEG channels.

### Data Analysis

#### Signal Pre-processing

The continuous ECoG time-series was down sampled at 500 Hz and re-montaged to a common average reference. Depth and grid electrodes were re-referenced and analyzed separately. Recordings were segmented into 400-ms pre-stimulus to 1000-ms post-stimulus trials. Channels and trials with excessive artifact, epileptiform activity or noise were excluded.

#### Auditory Responses

We used time-frequency, matching pursuit analysis to derive HG responses, defined as statistically significant (*p* < 0.05) increases in spectral power at 70–150 Hz relative to baseline (Mallat and Zhang, 1993; Franaszczuk et al., 1998). Significant changes in post-stimulus spectral power were determined by paired *t*-test, using log transformation and assuming unequal variances (Zygierewicz et al., 2005). False discovery rate correction was applied for multiple within-subject comparisons (Benjamini and Hochberg, 1995). Evoked response potentials (ERPs) were computed from the same ECoG signals using trial-averaging in the time domain.

To ensure only HG adapting sites were analyzed (Kar and Krekelberg, 2016), we used a normalized adaptation index (Ulanovsky et al., 2003; Eliades et al., 2014). HG response magnitude was based on estimated increases in log-transformed power density (decibel/hertz). Statistical testing by stimulus and electrode site was performed using non-parametric Wilcoxon rank sum test. Estimated log-power density values were compared using paired *t*-tests.

#### Adaptation Measurements

Single-trial measurements quantified the magnitude and time course of HG adaptation. Trial-to-trial changes were determined relative to the first frequent stimulus trial in each series and then averaged across sites for the first 50 frequent trials. Population single-trial responses were fit with a decaying exponential function to derive time constants of adaptation (Eliades et al., 2014).

#### Cross-Frequency Coupling

We used cross-frequency coupling to index local network activity (Buzsáki and Draguhn, 2004). We computed phase-amplitude coupling (PAC) between theta (4–7 Hz) and HG using the phase-locking value (PLV) method (Penny et al., 2008), defined as: PLV = 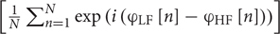 where φ_LF_[*n*] denotes the phase of theta (*LF*) and φ_HF_[*n*] denotes the high-gamma (*HF*) amplitude envelope. Theta is considered the main low-frequency modulator of HG (Buzsáki and Draguhn, 2004; Canolty et al., 2006). After filtering signals in theta and HG frequency bands, the PLV was calculated for the 400-ms post-stimulus period of each trial at all sites. PAC values were averaged across trials by position in each series of repeated frequents. Linear regression was fitted separately to depth and grid population-averaged PAC values. Correlation analysis was performed using Spearman rank correlations.

## Results

**FIGURE 1:**
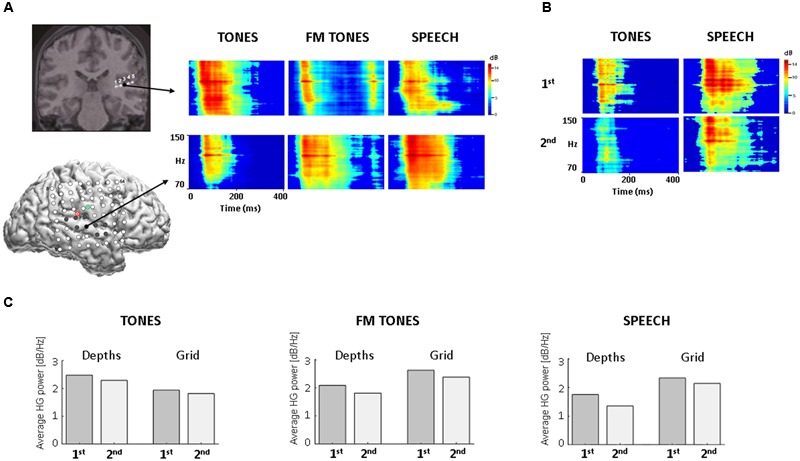
**HG spectral power as a function of stimulus and recording site in auditory cortex. (A)** Time-frequency plots of trial-averaged HG power for pure tones, FM tones, and speech, with time on the *x* axis (0–400 ms) and frequency on the *y* axis (70–150 Hz, high-gamma). (Top): coronal view from 3D MRI showing five co-registered contacts on the posterior depth (DP) electrode (for different view, see Supplementary Figure 1). Arrow projects from largest HG response sites (DP3, black filled). (Bottom): Sagittal view from 3D MRI showing location of co-registered grid electrodes on lateral right hemisphere (for larger view, see Supplementary Figure 1); red and green circles denote point of insertion for the posterior depth (DP, red) and anterior depth (DA, green). Filled electrodes denote locations of adapting HG responses. Arrow projects from largest HG response sites (black filled). **(B)** Time-frequency plots of HG power for the 1st and 2nd repetitions of tones (Left) and speech (Right) from the largest grid response site (RH27). Time-frequency plots are scaled to the high-gamma frequencies for improved visibility. **(C)** Bar graphs showing changes in average HG power by 1st and 2nd stimulus repetition at depth and grid sites.

Twelve HG adapting sites were identified: three posterior depth and nine grid sites. The three depth contacts (DP3-5) were contiguous and responsive to all stimuli, with pure tones eliciting the largest responses (**Figures 1A–C**). Imaging confirmed the locations of depth contacts in Heschl’s gyrus (**Figure 1** and Supplementary Figure 1A). Corresponding stereotactic coordinates (MNI, Talairach) mapped to primary auditory cortex (Supplementary Table 1). *Post hoc* comparisons by tone frequency showed larger responses to 1200 Hz vs. 1000 Hz (*p* ≤ 0.0059, Wilcoxon rank sum) at two depth contacts (DP3-4); the third contact (DP5) showed no significant differences (*p* = 0.224). No HG responses were elicited at the other two posterior depth contacts (DP1-2) or at the anterior depths (DA) located in inferior parietal cortex and insula.

High-gamma adapting sites on the grid localized mainly to superior temporal gyrus (*N* = 7) in auditory association cortex, as confirmed by imaging and stereotactic mapping (**Figure 1A** and Supplementary Table 1). Two additional sites were identified over the Sylvian fissure, partially overlapping the posterior superior temporal gyrus. The largest responses localized to the lateral posterior superior temporal gyrus. All HG sites responded to speech; fewer to FM tones (*N* = 7) and pure tones (*N* = 4). Visual inspection of time-frequency and population HG plots revealed smaller responses to repeated stimuli at both depth and grid sites, consistent with adaptation (**Figures 1B,C**).

Evoked (ERP) responses to frequent stimulus trials yielded reliable waveforms with robust N100 responses at all depth and grid sites where HG responses were observed in the same post-stimulus period. The largest N100 response at depths was for pure tones (DP4, -53.86 μV); the largest N100 at grid sites was for speech (RH28, -38.07 μV) (Supplementary Figure 2), consistent with the HG findings. To investigate ERP adaptation as a function of repetition, averaging by trial position was performed; however, the waveforms were too noisy to identify reliable responses likely reflecting the small number of trials in each position (<12 trials). Therefore, no further analysis of the ERPs was performed.

### Single Trial HG Results

**FIGURE 2:**
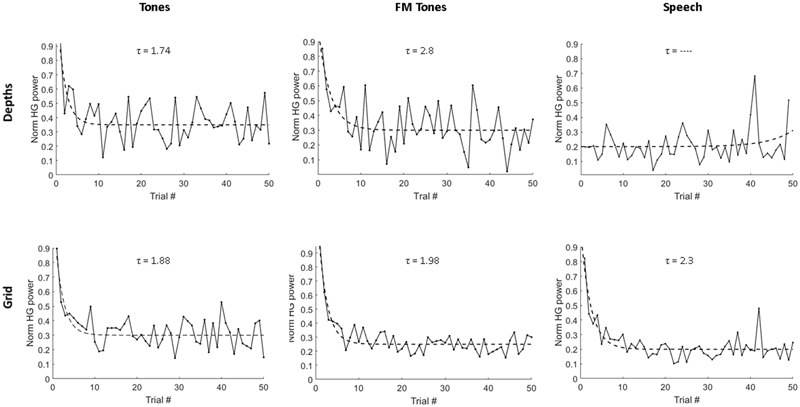
**Single-trial adaptation of HG power as a function of stimulus and recording site.** Plots show average HG response magnitude for depth electrodes **(Top)** and grid electrodes **(Bottom)** across first 50 repeated trials for pure tones, FM tones and speech. Dashed lines denote exponential fits used to derive adaptation time constants (τ), measured as number of stimulus repetitions and displayed above each plot.

Trial-to-trial decreases in HG magnitude were observed across sites and stimuli (**Figure 2**). Decreases in HG power reached steady-state levels by 4–5 repetitions, with the largest decreases occurring over the first 1–2 repetitions. Median adaptation time constants computed from exponential fits of population responses are shown in **Figure 2**. The fastest adaptation was for tones at depth sites (τ = 1.74); the slowest adaptation was for speech at grid sites (τ = 2.3). Adaptation rates for FM tones were slower than pure tones at the depths (τ = 2.8 vs. 1.74) and slower at depth versus grid sites (τ = 2.8 vs. 1.96 stimuli).

We also investigated whether the relatively large number of repeated infrequents (18%) may have triggered adaptation offsetting any response enhancement associated with deviance detection, as previously observed (Eliades et al., 2014). Single-trial measures of HG responses to infrequent FM tone and speech stimuli showed slow adaptation over the 300-trial run (3.3–4.5 stimulus repetitions) at grid sites. No decreases to infrequent stimuli were observed at depth sites. This suggests that adaptation in primary auditory cortex may be less sensitive to repetition over the long inter-stimulus separating infrequent stimuli (2.8–16.8 s) and could also account for the absence of adaptation to infrequent tones at grid sites. Additional studies varying inter-stimulus intervals are needed to test this possibility.

### Cross-Frequency Coupling Results

**FIGURE 3:**
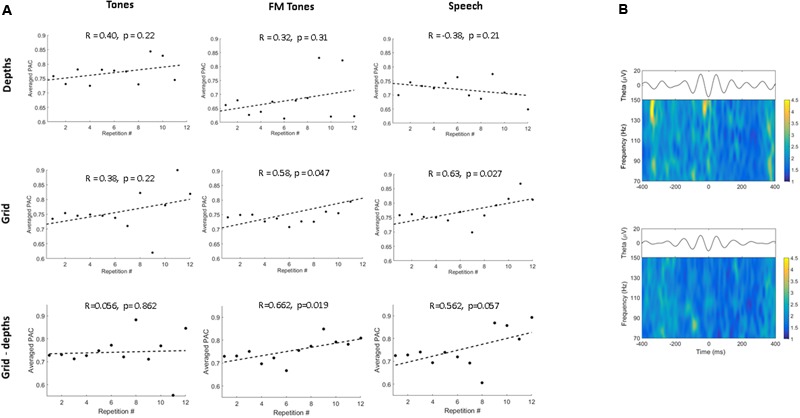
**Phase-amplitude coupling (PAC) as a function of stimulus and region. (A)** Correlation plots show population theta-to-high gamma PAC trends as a function of repetition for pure tones, FM tones, and speech. PAC values are averaged across the first 12 stimulus repetitions. PAC trends are shown by region for depth (Top) and grid sites (Middle) and between regions (inter-regional, depth-grid) (Bottom). Dashed lines denote linear regression fitted to trends of averaged PAC values. Correlation coefficients and *p*-values are displayed above each plot. **(B)** Time-frequency plots showing mean high-gamma power modulation time-locked to the theta phase trough (plotted above) for speech for one grid electrode in association auditory cortex (Top) and one depth electrode in primary auditory cortex (Bottom).

To investigate repetition effects on local network activity, we measured PAC of theta and HG at depth and grid sites. **Figure 3A** shows population PAC trends by stimulus and region. For pure tones, PAC showed similar, non-significant trends at depth and grid sites. For FM tones, PAC increased in both regions, but was significant only at grid sites (*p* = 0.047). For speech, PAC values only increased at grid sites (*p* = 0.027).

Repetition effects on inter-regional PAC between sites in primary and association auditory cortex showed no significant increases (*p* = 0.86) for tones (**Figure 3**). Conversely, PAC increased between primary and auditory association cortex for FM tones (*p* = 0.019) and speech (*p* = 0.057). To determine whether PAC trends were specific to theta (4–7 Hz), PAC values were re-computed using alpha band frequencies (8–13 Hz). Results showed no repetition-related increases or consistent PAC trends for alpha (Supplementary Figure 3).

Although no auditory-related HG responses were observed in the frontal lobe, a *post hoc* PAC analysis was performed for inferior frontal lobe sites to investigate potential top-down effects. Within-region PAC computed for three sites in the inferior frontal lobe showed no repetition-related increases by stimulus (*p* ≥ 0.27). Similarly, no inter-regional PAC trends were observed between inferior frontal (theta) and auditory association sites (*p* ≥ 0.26) or primary auditory sites (*p* ≥ 0.29) (Supplementary Figure 4). The only inter-regional PAC trend that reached significance (*p* = 0.048) was negatively correlated for FM tone repetition.

### Surgical Outcome

Clinical ECoG recordings localized the patient’s seizures to the inferior parietal dysplasia. He underwent tailored focal resection, sparing auditory cortex. No post-operative deficits were reported; the patient demonstrated normal speech understanding in background noise on retesting 4 months after surgery. The patient’s complex partial seizures resolved after surgery and he remained seizure free at the last 5-year follow-up visit.

## Discussion

Simultaneous ECoG recordings from primary and association auditory cortex revealed repetition effects that were stimulus- and region-specific. For pure tones, we observed similar patterns of HG adaptation and PAC in both cortical regions, suggesting a single origin in primary auditory cortex. Depth contacts in Heschl’s gyrus showed greater adaptation for pure tones and frequency selectivity, consistent with tonotopic organization of human primary auditory cortex as first shown in a seminal depth recording study by Howard et al. (1996).

FM tones and speech sounds elicited HG responses in both primary and association auditory areas, consistent with prior studies (Brugge et al., 2009; Nourski et al., 2013), and showed distinct regional patterns of adaptation. We observed slower adaptation for FM tones in primary auditory cortex compared with auditory association cortex. Adaptation might be slower in primary auditory cortex if each component frequency is processed sequentially along the tonotopic gradient, whereas auditory association cortex also receives input from non-lemniscal thalamic pathways (Kaas and Hackett, 2000) and, therefore, may initiate adaptation earlier. Alternatively, slower adaptation to FM tones in primary auditory cortex could reflect feedback from auditory association cortex (Kumar et al., 2011). Because ECoG recordings are limited to cortical activity, it was not possible to differentiate between these two possibilities.

HG adaptation to speech was observed only in auditory association cortex, suggesting that the lateral temporal lobe plays a role in suppressing repetitive, complex sounds. This has potential clinical implications for patients undergoing right temporal lobe resections as they may have increased listening difficulties in real-world environments where repetitive background sounds (noise) are common (Drew et al., 2003; Nagle et al., 2013).

Significant increases in PAC were observed in auditory association cortex for FM tones and speech, but not pure tones. This finding is consistent with our single-trial results and suggests that tone adaptation occurs mainly in primary auditory cortex, whereas adaptation to complex sounds occurs in auditory association cortex. Repetition-related increases in inter-regional PAC were also observed for speech and FM tones but not for pure tones, suggesting multiple, stimulus-dependent sources of theta modulation in auditory cortex. Substituting alpha frequencies yielded no increases in PAC, indicating that phase-modulation of HG may also be theta specific.

Taken together, these results suggest that adaptation in human auditory cortex during passive listening has multiple stimulus-dependent origins, consistent with hierarchical models of cortical auditory processing (Boatman et al., 1995; Rauschecker et al., 1995; Kaas and Hackett, 2000). Although we did not observe PAC increases at frontal lobe sites, this does not preclude top-down modulation (feedback) of auditory cortex under other auditory conditions, such as attended listening, or using other connectivity methods. Results from a single subject with limited spatial sampling also preclude generalization and require verification with larger studies.

## Concluding Remarks

Our results suggest that adaptation in human auditory cortex has multiple regional and inter-regional origins. Neural mechanisms of adaptation appear sensitive to stimulus complexity, consistent with hierarchical cortical auditory models. These results have implications for neuroimaging studies using adaptation paradigms as stimulus factors may determine sources of sub-regional functional selectivity. Our results reveal a novel functional network for adaptation involving the non-dominant temporal lobe. These results underscore the need for accurate localization to avoid potential post-operative deficits, including previously unrecognized difficulty suppressing repetitive background sounds (noise) in everyday listening environments.

## Ethics Statement

The procedures were approved by the Johns Hopkins Institutional Review Board. Written informed consent in accordance with Johns Hopkins Institutional Review Board requirements. The research had no impact on clinical care of the patient.

## Author Contributions

DB-R designed the study. FL performed the surgery. NC and DB-R collected the data. UM, DB-R, and MC analyzed the data. UM and DB-R wrote the manuscript. UM, NC, FL, and MC revised the manuscript. All authors approved the final version of the manuscript.

## Conflict of Interest Statement

The authors declare that the research was conducted in the absence of any commercial or financial relationships that could be construed as a potential conflict of interest.
