# Supplementary material for: Multi-Regional Adaptation in Human Auditory Association Cortex
Source: Front Hum Neurosci. 2017 May 9;11:247. doi: 10.3389/fnhum.2017.00247 (PMC5422464; doi:10.3389/fnhum.2017.00247)
Supplement: Supplementary file 1 [file Data_Sheet_1.docx]

Supplementary Material

**Multi-Regional Adaptation in Human Auditory Association Cortex**

Urszula Malinowska, Nathan E. Crone, Frederick A. Lenz, Mackenzie Cervenka, Dana Boatman-Reich^*^

*** Correspondence:** Dana Boatman-Reich: [dboatma@jhmi.edu](mailto:dboatma@jhmi.edu)

# Supplementary Table

**Table S1**. Stereotactic coordinates and corresponding Brodmann areas for HG adapting depth and grid electrode sites in the right hemisphere.

| Electrode | Talairach Coordinates  (x,y,z) | Brodmann Number & Area |
| --- | --- | --- |
| Depth electrode DP3 | 39, -28, 11 | BA 41, primary auditory |
| Depth electrode DP4 | 44, -26, 12 | BA 41, primary auditory |
| Depth electrode DP5 | 49, -25, 14 | BA 41, primary auditory |
| Grid electrode RH 12 | 57, -39, 12 | BA 22, auditory association |
| Grid electrode RH 19 | 57, -26, 4 | BA 22, auditory association |
| Grid electrode RH 20 | 58, -29, 13 | BA 22, auditory association |
| Grid electrode RH 27 | 58, -17, 5 | BA 22, auditory association |
| Grid electrode RH 28 | 61, -20, 8 | BA 22, auditory association |
| Grid electrode RH 34 | 55, -6, -4 | BA 22, auditory association |
| Grid electrode RH 42 | 52, -2, 4 | BA 22, auditory association |
| Grid electrode RH 35 | 54, -9, 13 | BA 1, inferior parietal (primary sensory) |
| Grid electrode RH 43 | 52, -1, 17 | BA 6, inferior parietal (pre-motor) |

# Supplementary Figure


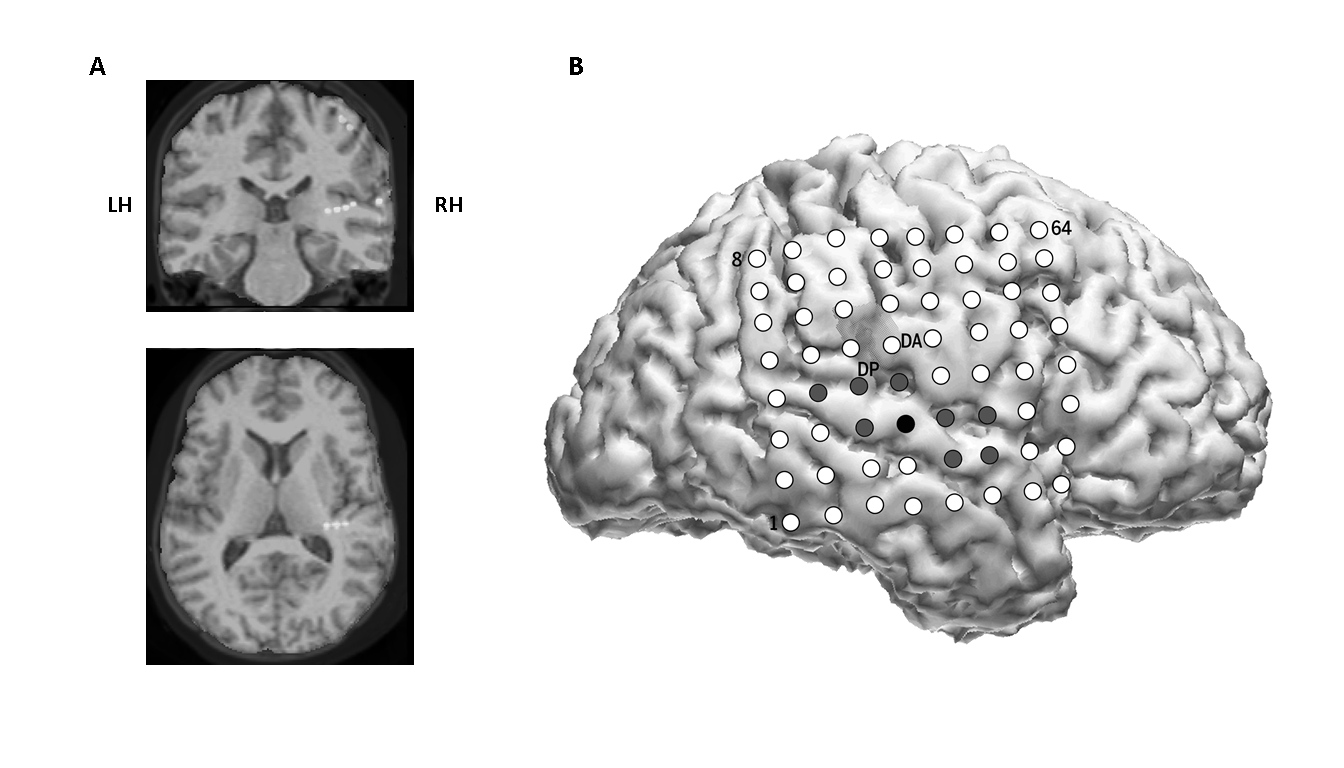


**Supplemental Figure 1**. **MRI images with co-registered electrodes**. **(A)** Coronal (top) and corresponding axial (bottom) views showing location of co-registered posterior depth electrode (DP) contacts. Contact DP3 which showed the largest auditory responses is visualized on both views. Four contacts are visible on the coronal view corresponding to DP 4-1 in descending order from lateral to medial; *Note*: the most lateral of the five depth contacts (DP5) is not visualized on this coronal slice. The axial view shows the three most medial contacts: DP3-1 in descending order from lateral to medial. **(B)** Sagittal 3D MRI view of lateral right hemisphere showing co-registered grid electrodes. Electrode numbering starts with left-most electrode on bottom row (#1) and ends with right-most electrode on top row (#64). Depth insertion points are designated by DP (posterior depth) and DA (anterior depth). Gray mesh region in inferior parietal lobe denotes location of cortical dysgenesis.

**
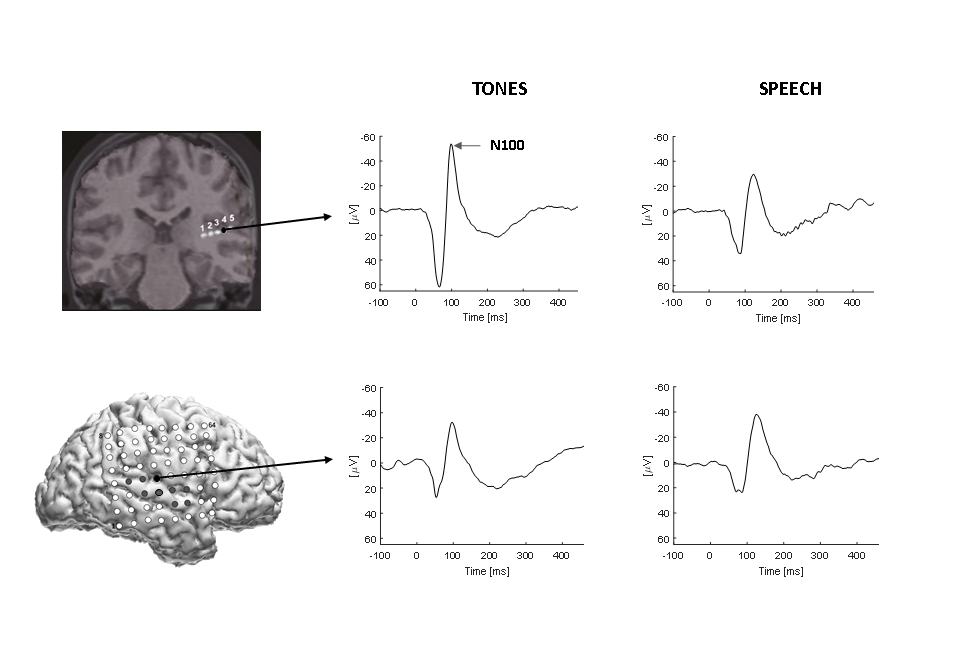
**

**Supplemental Figure 2. Evoked (ERP) responses as a function of stimulus and recording site.** Top: MRI coronal image showing co-registered posterior depth electrode contacts; arrow from black-filled electrode denotes site of largest ERP responses to frequent tone (left plot) and speech stimuli. The evoked N100 response is labelled in the tone ERP plot, with negative peak pointing upwards. Bottom: Lateral view of right hemisphere showing co-registered grid electrode locations. Arrow from black-filled electrode denotes site of largest N100 response to tones (left) and speech. Gray-filled electrodes represent additional ERP sites. For ERP plots, time is on the x-axis in milliseconds; amplitude is on the y-axis in µV, with negative values above the zero line.

**
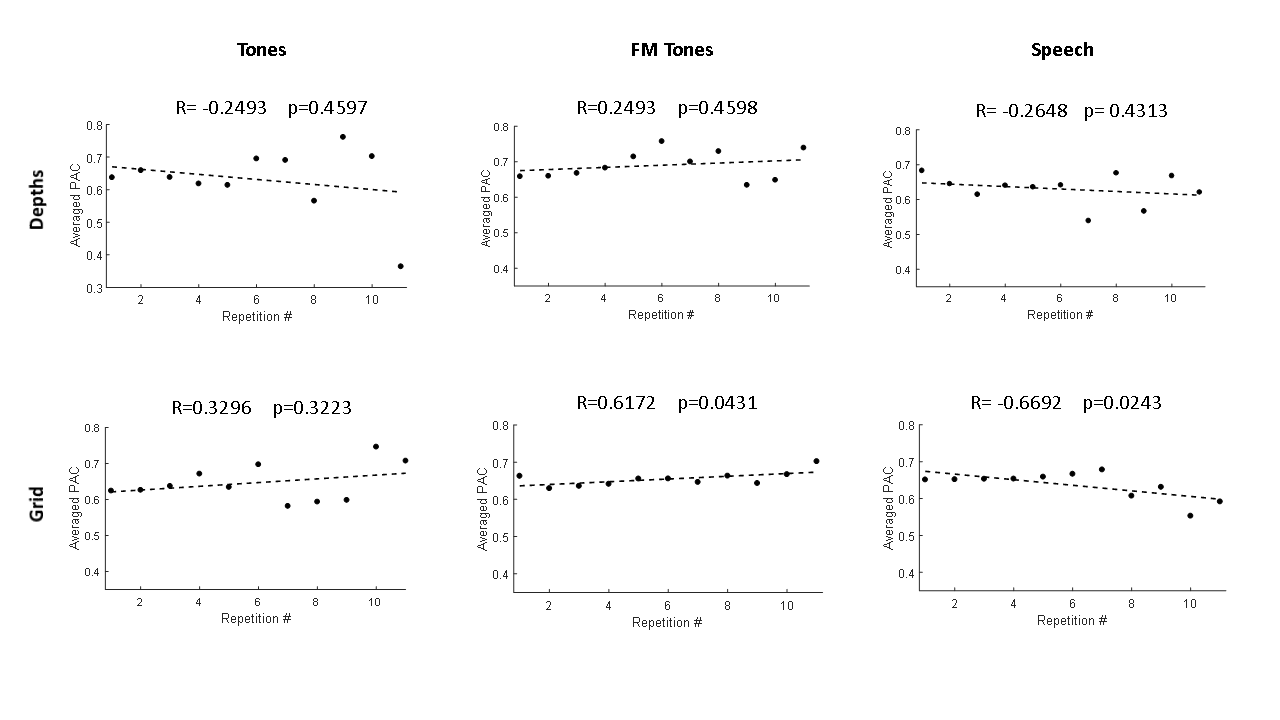
**

**Supplemental Figure 3. Phase-amplitude coupling (PAC) between alpha and high gamma as a function of stimulus and recording site**. Correlation plots show population alpha-to-high gamma PAC trends as a function of stimulus repetition. PAC values are averaged across the first 12 stimulus repetitions for depth (top row) and grid sites (bottom row) for comparison with theta-to-high gamma trends shown in Figure 2. Dashed lines denote linear regression fitted to averaged PAC values. Correlation coefficients and p-values are displayed above each plot.

**
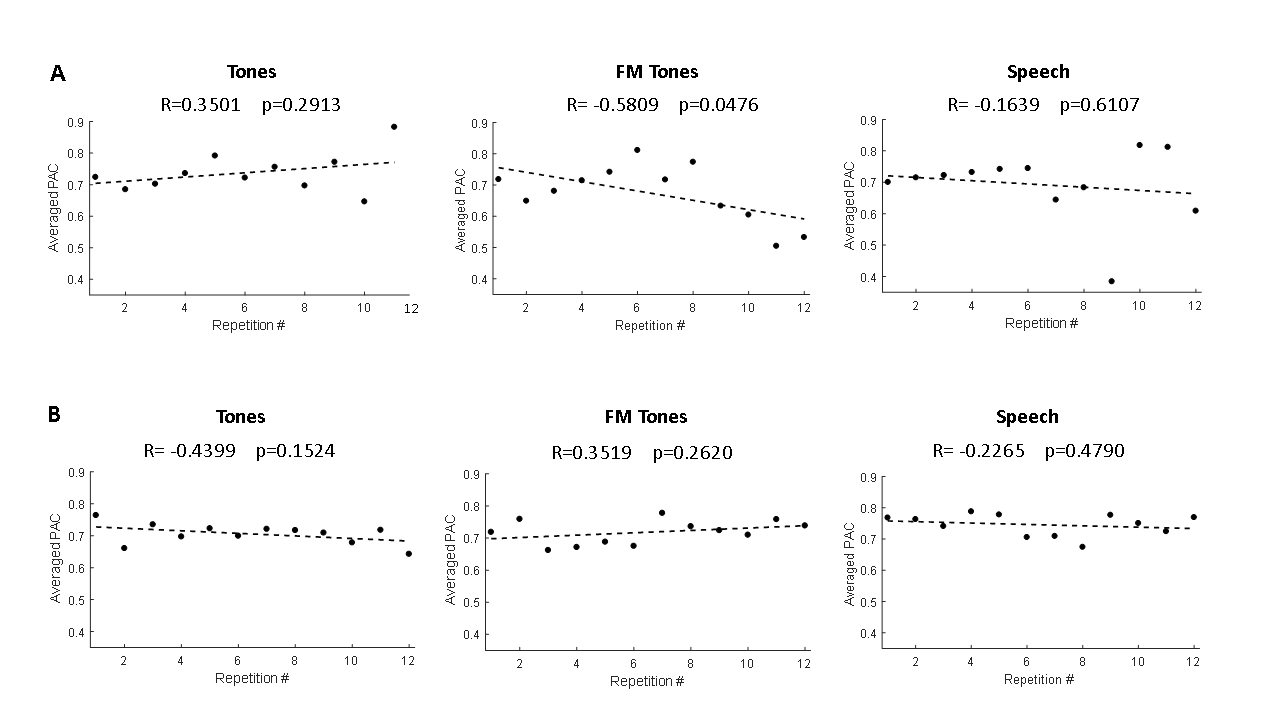
**

**Supplemental Figure 4. Cross-frequency coupling between inferior frontal lobe and auditory cortex. (A)** Correlation plots show population PAC trends between inferior frontal and primary auditory sites as a function of stimulus repetition. **(B)** Correlation plots show PAC trends between inferior frontal and auditory association sites as a function of stimulus repetition. PAC values are averaged across the first 12 stimulus repetitions. Dashed lines denote linear regression fitted to trends of averaged PAC values. Correlation coefficients and p values are displayed above each plot.
